# A Roadmap for Migrating Neurons

**DOI:** 10.1371/journal.pbio.0060153

**Published:** 2008-06-10

**Authors:** Caitlin Sedwick

Politicians, pundits, and even your best friends occasionally do things that make you wonder how their brains are wired. The next time you have that thought, consider consulting a developmental neuroscientist: they work every day to understand the processes that wire up *everyone's*brains. It's a mind-boggling job, because as an embryo develops, the connections within its brain ramify, becoming ever more complex. For example, consider the neural connections in the mammalian cerebellum. This distinctive structure is responsible for coordinating sensory and motor signals to produce fine motor control. This must be a complicated job—the cerebellum contains about half of the brain's neurons.

The cerebellum receives many of its inputs from pontine neurons, which in adults are located in an area of the brain adjacent to the cerebellum itself. But embryonic pontine neurons are generated in a part of the brain far from the cerebellum, so they must undertake a migration to reach their ultimate destination. In mice, pontine neurons follow a very strict route: they migrate from the rear of the brain forward until they reach the front of the brainstem, and then they take a sharp turn toward the bottom surface of the brain stem. The processes guiding pontine neurons on this path are mainly a mystery but, as Marc Geisen, Filippo Rijli, and colleagues show in a new study, it's now clear that the route followed by pontine neurons is dictated in large part by *Hox* genes.

*Hox* genes express a family of transcription factors better known for their role in anterior-posterior segmental body patterning. In insects, mis-expression of *Hox* genes can cause insects to grow legs in place of antennae. More importantly for this work, *Hox* genes also direct hindbrain segmentation in mammals. Because of their prominent role in conveying positional information to cells during development, Geisen et al. felt that *Hox* genes were likely to help guide mammalian pontine neurons on their long trek. Therefore, they decided to examine which *Hox* genes are expressed in mouse pontine neurons during migration from the site of their origin to their final destination.

**Figure:**
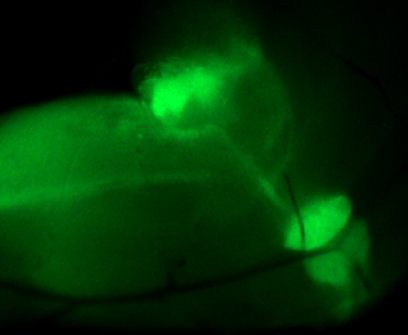
In this whole-mount lateral view of an embryonic day 17.5 mouse brain, an *EGFP *knock-in allele into the *Hoxa2 *locus allows direct visualization of pontine neurons during their migration to their final destination in the anteroventral brain stem.

The researchers first showed that embryonic pontine neurons express several *Hox* genes, and they targeted two of these genes, *Hoxa2* and *Hoxb2*, for further study. They found that knocking out expression of *Hoxa2* (or, to a lesser extent, *Hoxb2*) caused some pontine neurons to prematurely divert toward the bottom surface of the brain stem. It turns out that *Hoxa2* controls this behavior by modulating pontine neurons' responsiveness to chemoattractive and chemorepellant signals in their environment.

To help visualize this, imagine the situation faced by a sleep-deprived motorist driving down a two-way city road crammed with cafés—all on the opposite side of the street. Although the motorist might badly want to cross the road to get some java, the double-yellow line makes such a move illegal. But a break in the painted barrier gives the driver the go ahead to finally get some relief.

Migrating pontine neurons are being lured by a chemoattractant (as tempting as good coffee) produced by the cells that line the bottom surface of the brain stem. The authors' experiments show that pontine neurons are prevented from prematurely heading toward the attractant by a repulsive chemical called Slit, which is produced by cells of the facial motor nucleus, a structure that lies alongside the migratory path. Slit binds to the receptor Robo (which is expressed on the surface of pontine neurons) and prevents the neurons from responding to chemoattractants. The authors suggest that once the pontine neurons have migrated past the facial motor nucleus, the Slit signal wanes in intensity, providing a break in the repulsive barrier. This lets pontine neurons move toward the chemoattractant.

The reason *Hoxa2*-deficient brains show aberrant pontine neuron migration is that *Hoxa2* directly controls Robo expression in pontine neurons by binding to Robo DNA, and it indirectly controls Slit expression levels in the facial motor nucleus. Loss of *Hoxa2* therefore causes some pontine neurons to become insensitive to Slit signaling, letting them ignore the repellant signal and head prematurely toward the chemoattractant. This conclusion is reinforced by the finding that if Slit or Robo (or the facial motor nucleus) is absent during development, pontine neurons migrate abnormally, similar to *Hoxa2* knockouts.

Interestingly, since not all pontine neurons lose their inhibitions when *Hoxa2* is lost, it seems likely that other factors (possibly other *Hox*genes) may be involved in adjusting the balance of attractive and repellant responses. This idea remains to be explored in the future, but these findings have already started to reveal the route pontine neurons follow in their migration.

